# Infection of bats with *Histoplasma* species

**DOI:** 10.1093/mmy/myad080

**Published:** 2023-08-08

**Authors:** Harish C Gugnani, David W Denning

**Affiliations:** Professor of Medical Mycology (Retired), Department of Microbiology, Vallabhbhai Patel Chest Institute, University of Delhi, Delhi, India; Manchester Fungal Infection Group, School of Biology, Medicine and Health, University of Manchester, Manchester Academic Health Science Centre, Manchester, UK

**Keywords:** histoplasmosis, cave, outbreak, latency, *Histoplasma. capsulatum* var. *duboisii*

## Abstract

*Histoplasma* species infect humans and animals, notably bats. *Histoplasma* species are thermally dimorphic fungi existing in mycelial form in the natural environment and in yeast form in infected tissues. In this narrative literature review, we summarize the occurrence of *Histoplasma* spp. in different species of bat tissues (*n* = 49) and in soil admixed with bat guano where the species of bat dwelling nearby has been identified (an additional 18 species likely infected) to provide an up-to-date summary of data. Most positive isolations are from the Americas and Caribbean, with some studies from Thailand, Malaysia, Nigeria, Slovenia, France, and Australia. We also summarize some of the early experimental work to elucidate pathogenicity, latency, immune response, and faecal excretion in bats. Given the recent recognition of the global extent of histoplasmosis, thermal dimorphism in *Histoplasma* spp., and global heating, additional work on understanding the complex relationship between *Histoplasma* and bats is desirable.

## Introduction

A genus of pathogenic fungi shared between humans and bats is *Histoplasma* spp. Initial work on the natural habitat of *Histoplasma* spp. focused on chicken houses in the 1940’s and 1950’s.^1,2^ The first link to bats came from positive cultures from soil in a bat cave in Venezuela in 1956,^3^ and then from soil in South Africa, Tanzania, and Trinidad.^4–6^ The first isolation from a bat (*Chilonycterus rubiginosa fusca*) came from Panama in 1962.^7^*Histoplasma duboisii* has only been isolated once from bats in Nigeria.^8^

*Histoplasma capsulatum* is a thermally dimorphic saprobic fungus that produces microconidia and macroconidia borne on mycelium.^9,10^ When suitable microclimatic conditions of temperature (22°C–29°C) and annual rainfall of 35–50 inches are present, and soil is enriched with avian excreta or bat guano, the fungus proliferates abundantly. Birds don't get infected with *H. capsulatum*, whereas bats can be infected and can disperse the fungus creating new foci of infection.^11^ Infection is acquired by inhalation of microconidia or hyphal fragments of *H. capsulatum* from environmental sources such as soil or dried avian or bat excreta. The rapidly changing global environment will inevitably alter some of these interactions.

Following inhalation by a human, the infectious propagules of *H. capsulatum* are engulfed by pulmonary macrophages, wherein they convert to the yeast form of the fungus and multiply and traverse to hilar and mediastinal lymph nodes.^9,10^ The yeast form of the fungus then gets access to the blood circulation system and disseminates swiftly across various organs of the body. After approximately two weeks of exposure, the macrophages become fully fungicidal, and cellular immunity increases, with defense against the fungal particles.^10^ This process can lead to necrosis at the sites of infection in the lungs, liver, spleen, lymph nodes, bone marrow, adrenal glands, and mucocutaneous membranes, and acute, sub-acute, or chronic disseminated histoplasmosis, acute pulmonary histoplasmosis, or latency. The human pathogenic life cycle of *H. duboisii* is less well understood.^12,13^ The full life cycle of *H. capsulatum* has been partially elucidated in experimental bat infections.^14–17^

Investigations by several workers have shown that bat guano and bird droppings are the most common sources of *Histoplasma capsulatum*, the etiological agent of histoplasmosis, which has a worldwide distribution.^11,18–20^ The fungus is found either in spaces where bat guano is abundant or in open spaces such as public parks and home yards, where avian droppings are frequently found. Bat and avian excreta provide nutrients necessary for fungal growth and with soil and environmental conditions, humidity, and temperature, constitute the ecologic niche of this microorganism.^1,19,21^ Bats are among the few infected mammals that contribute to the maintenance of this fungus in natural foci. Our aim was to review the species of bats that have been reported to be infected by *Histoplasma* spp. and to link those reports with the bats’ locations. The last time this topic was reviewed was in 1981 by Hoff and Bigler.^18^

## Materials and Methods

An exhaustive search of the literature, using PubMed, MEDLINE, Med Facts, and LILACS (a database Latin American and Caribbean Health Sciences Literature) was made for all years to September 10^th^ 2022 using different sets of keywords, viz. histoplasmosis, bats, caves, *Histoplasma capsulatum* var, *capsulatum, Histoplasma capsulatum* var. *duboisii*. The Boolean operator ‘AND’ was used for combining and narrowing the searches. Cross-references were also accessed to extract information relevant for incorporation into the review. Review of papers that were identified in searches also identified references that were not listed online and these were retrieved. No language restriction was applied.

## Results

We identified 45 articles describing the isolations of *Histoplasma* spp. from bats, or detection using histopathology, polymerase chain reaction (PCR) or antigen. A smaller number or articles linked specific bat species to outbreaks in humans, providing inferential evidence of individual bat species infection. The data collected and the important findings found in the survey of literature are described below under different subheadings.

### Occurrence of *Histoplasma duboisii* in bats and environmental sources

The only bat isolation of *Histoplasma duboisii* is from Nigeria in *Nycteris hispida* (hairy slit faced bat with long ears) and *Tadarida pumila* (little free-tailed bat) (Table 1). This was from intestinal contents only of a single bat, but it is not clear which species. Histological examination of the tissue of liver, spleen, and lung from this particular bat and 8 additional bats of *Nycteris hispida* and *Tadarida pumila* did not reveal the presence of *Histoplasma* nor were the cultures from the internal organs of any of the bats examined positive for *Histoplasma* yeast forms. It is therefore unclear whether this fungus can infect bats.

**Table 1.** Association of *H. duboisii* with bats.

Country	Location	Sample type, No. pos./no. examined	Method of detection	Reference
Nigeria	Ogbunike, Anambra State	Soil admixed with bat guano in a cave. 8/45 were positive.	Culture on mycological media and by intraperitoneal mouse inoculation	Gugnani *et al*. 1994^8^
Nigeria	Ogbunike, Anambra State	Intestinal contents of bats of the species *Nycteris hispida* (hairy slit-faced bat) and *Tadarida pumila* (little free-tailed bat). 1/35 positive from all tested.	Direct plating of serial dilutions of antibiotic treated suspension of soil/bat guano on fungal media supplemented with chloramphenicol and/or cycloheximide. Intraperitoneal inoculation of mice with each suspension. Intestinal contents of 13% of the bats were positive for exoantigen of *H. duboisii*.	Gugnani *et al*. 1994^8^

### Isolation of *Histoplasma capsulatum* directly from bats

We have tabulated the species of bats from which *H. capsulatum* has been directly isolated (Table 2; Supplementary Table 1). We found direct evidence of isolation from bat tissues in 48 species, all but one from the Americas and Caribbean, in studies from 1962 to 2021. Some bat species were sampled and cultures were negative (Supplementary Table 1). Attempts to isolate *H. capsulatum* from bats in Iran^44^ and Zaire (now Democratic Republic of Congo)^45^ were negative. In some studies, the bat species was not determined.

**Table 2.** Bat species and country from which *Histoplasma capsulatum* has been isolated. This excludes soil and guano samples, even if the roosting bat species was identified.

Species	Common name and additional data	Countries	References
*Artibeus hirsutus*	Hairy fruit-eating bat only known in Ecuador and Peru	Mexico	Taylor, 1999^22^; Taylor, 2012^23^
*Artibeus fimbriatus*	Fringed fruit-eating bat	Brazil	Veloso, 2014^24^
*Artibeus jamaicensis parvipes*	Hairy fruit-eating bat endemic in Mexico	Cuba	D'Escoubet, 1976^25^
*Artibeus literatus*	Great fruit-eating bat	Brazil	Veloso, 2014,^24^ Dos Santos, 2018^26^
*Brachyphylla nana*	Cuban fruit-eating bat found in Cayman Islands and Hispabiola. Prefers to live in deep caves that are often hot and humid	Cuba	D'Escoubet, 1976^24^
*Carollia perspicillata*	Short-tailed bat found in Central America	Colombia, Panama, Brazil	Klite, 1965^27^; Diercks, 1965^28^; Tesh, 1968^29^; Shacklette, 1969^30^; Hasenclever, 1972^31^ da Silva 2020^32^
*Chilonycterus rubiginosa fusca* *Pteronotus parnellii* *Pteronotus rubiginosa*	Found in flowering trees including purple coral tree Parnell's mustached bat Wagner's Common Mustached Bat	Panama, Belize, Mexico	Shacklette, 1962^7^; Klite, 1965^27^; Diercks, 1965^28^; Shacklette, 1967^33^; Shacklette, 1969^30^; Hasenclever, 1972^31^; Quinones, 1978^34^; Taylor, 1999^22^
*Desmodus rotundus*	Small leaf-nosed vampire bat native to neotropics	Colombia, Brazil	Diercks, 1965^28^; Tesh, 1968^29^; Shacklette, 1969^30^; Taylor, 2012^23^ Veloso, 2014^24^
*Eptescicus brasiliensis*	Brazilian brown bat found in neotrpics	Colombia, USA	Tesh, 1968^29^; Emmons, 1966^35^
*Eptesicus fuscus dutertreus*	Big brown bat commonly found in in heavily forested regions	Cuba	D'Escoubet, 1976^25^
*Eumops bonariensis*	Dwarf bonneted bat, or Peters' mastiff bat found in leaved dry grasslands.	Brazil	Taylor, 2012^23^
*Eumpos glaucinus*	Wagner's bonneted bat, found in subtropical forests	Brazil	Veloso, 2014^24^; Dos Santos, 2018^26^
*Eumops perotis*	Western mastiff bat	Brazil	Dos Santos, 2018^26^
*Glossophaga soricina*	House bat Pallas's long-tongued bat	Panama, Brazil	Klite, 1965^27^; Diercks, 1965^28^; Shacklette, 1969^30^; Hasenclever, 1972^31^ Veloso, 2014^24^
*Histiotus velatus*	Tropical big-eared brown bat	Brazil	Velosos, 2014^24^
*Lasiurus blossevillii*	Southern red bat	Brazil	Velosos, 2014^24^
*Leptonycteris curasoae*	Southern long-nosed bat found in semiarid and dry forests	?	Taylor, 2012^23^
*Leptonycteris nivalis*	Greater long-nosed bat living in desert scrub areas	Mexico	Taylor, 1999^22^; Taylor, 2012^23^; Vite-Garin, 2021^36^
*Leptonycteris sanborni*	Sanborn's long-nosed bat found in caves, rock crevices, old buildings, bridges, mines and trees	USA (Arizona)	Di Salvo, 1969^37^
*Lonchophylla aurita*	Tomes's or common sword-nosed bat found in forested and sometimes in agricultural area	Panama	Diercks, 1965^28^; Shacklette, 1969^30^
*Lonchophylla robusta*	Orange nectar bat found in some South American counties	Panama	Shacklette, 1969^30^
*Micronycteris megalotis*	Little big-eared bat found in wet and dry areas, evergreen forests, and swamps throughout South America	Panama	Klite, 1965^27^
*Molussus sp.r*	Mastiff bat species	Panama	Shacklette, 1969^30^
*Mollosus major*	Velvety free-tailed batthat occurs in Central and South America. Also found roosting in houses in urban areas	Panama	Klite, 1965^27^; Hasenclever, 1972^31^
*Mollosus mollosus*	Pallas’ mastiff bat found in subtropical forests and fields throughout Central and South America	Argentina, Brazil	Taylor, 2012^23^ dos Santos, 2018^26^ Veloso, 2014^24^
*Mollosus rufus* (*Molussus afer*)	Black mastiff bat found throughout Central and South America. Can be found in dark places like rock fissures, in caves, roofs and cracks of buildings	Mexico, Brazil	Taylor, 2012^23^ Veloso, 2014^24^
*Mormoops blainvillei*	Antillean ghost-faced bat found in caves throughout the world	Cuba	D'Escoubet, 1976^25^
*Mormoops megalophylla*	Ghost-faced bat typically found near desert shrubs in Central and South America	Mexico	Taylor, 1999^22^
*Myotis austroriparius*	Southeastern myotis feeds on a variety of insects, living close to water sources	Florida, USA	Tesh, 1967^38^; Hoff, 1981^18^
*Myotis californicus*	Californian myotis found in caves, mines, rocky hill sides, under tree bark, on the ground, and in buildings	Mexico	Taylor, 1999^22^
*Myotis grisescens*	Gray bat that occurs in a limited range in limestone areas of Southeastern USA	USA	Tesh, 1967^38^
*Myotis levis*	Yellowish myotis	Brazil	Veloso, 2014^24^
Myotis lucifugus*	Little brown bat often uses human made structures, typically roots in caves and mines in winter, and under rocks and piles of wood in summer	USA	Bell cit Emmons, 1966)^35^
*Myotis nigricans*	Black myotis	Brazil	Veloso, 2014^24^
*Myotis sodalis*	Indiana bat occurs in arid habitats, but can use a wide range of roosts	USA	Tesh, 1967^38^
*Natalus stramineus*	Gray funnel-eared bat, native to the Nearctic and the Neotropics	Mexico	Taylor, 1999^22^
*Noctilio labialis*	Lesser long-nosed bat, roosts externally on tree trunks or in the branches of trees	Panama	Shacklette, 1969^30^
*Nyctalus noctula*	Common noctule generally resides in forests, most often found in lowland areas and those with old forests and rivers	France	Gonzalez-Gonzalez, 2013^39^
*Nyctinomops laticaudatus*	Broad-eared bat or broad-tailed bat	Brazil	Veloso, 2014^24^
*Nyctinomops macrotis*	Big free-tailed bat	Brazil	Veloso, 2014^24^
*Phyllostomus discolour*	Pale spear-nosed bat found in mature and secondary rain forest, dry forest, gardens and plantations	Panama, El Salvador	Shacklette, 1969^30^; Klite, 1965^40^
*Phyllostomus hastatus*	Greater spear-nosed bat occurs in tropical regions of America	Panama	Klite, 1965^27^; Shacklette, 1969^30^; Hasenclever, 1972^31^
*Platyrrhinus lineatus*	White-lined broad-nosed bat	Brazil	Dos Santos, 2018^26^
*Pteronotus suratensis*	Taxonomy changed. Mustached bat	Panama	Shacklette, 1969^30^
*Tadarida brasiliensis* *T. brasilensis cynocephala*	Mexican free-tailed bat occurs in caves, mine tunnels, old wells, hollow of trees	USA, Mexico, Argentina, Brazil	Ajello, 1967^41^; Bryles, 1969^42^; Emmons, 1966^35^; Gonzalez-Gonzalez, 2012^43^; Taylor, 2012^23^; Veloso, 2014^24^; Vite-Garin, 2021^36^
*Tadarida yucatanica*	Broad-eared bat occurs in tropical and subtroical forests from coastal Mexico to southern Brazil	Panama	Shacklette, 1969^30^; Hasenclever, 1972^31^
*Tonatia biden*	Northern long-eared bat	Panama	Shacklette, 1969^30^
*Vampyriscus bidens*	Bidentate yellow-eared bat	Brazil	Da Silva, 2020^32^

* presumptive identification

These mostly culture-based studies have almost always included analysis of liver and spleen, usually lungs and sometimes intestines and/or intestinal contents. One study of 122 bats randomly captured in Argentina, French Guyana, Mexico found 77% positive by PCR of the lungs only,^46^ and a single *Nyctalus noctule* bat in France was also positive by PCR from lungs.^38^ The assumption is that if bat tissues are positive, then the bat is infected. Given the pathology of the intestine showing clumps of yeast and concurrent positive cultures from the intestine demonstrated in *Pteronotus* (*Chilonycteris) rubiginosa*,^28,47^ this seems a reasonable assumption given the diets of bats. Some bats have histological evidence of infection in various tissues, with negative cultures, others positive cultures, without evidence of fungi histologically.^44^ Such studies have only been done in a limited number of bat species. The relative proportion of organs that were culture positive in several species of bats captured in SE USA (primarily *Myotis austroriparius* and *Myotis grisescens*) were lungs (88%), spleen (73%), liver (72%), and faeces (36%).^38^ Recent studies from Brazil have limited sampling to the lungs and only used PCR for detection.^24,26,32^

Experimental infections using intraperitoneal inoculation demonstrated mice to be infectable with very few or a single spore^14,48^ and bats were experimentally infected via both intranasal and intraperitoneal routes with inocula as low as 10 infective units intraperitoneally and 100 intranasally.^15–17,46^ Experimental infection found differences between species. So *Tadarida brasiliensis* had an 80% mortality whereas *Artibeus lituratus* and *Pteronotus suapurensis* were infectable, but without any mortality.^18^ Experimental intranasal infection of the lungs of *A. lituratus* was possible with fewer than 100 viable spores,^49^ and led to disseminated infection, antibody production, and temporary cutaneous delayed hypersensitivity. Subsequently yeasts were shed via the intestinal villi into the faeces. Naturally infected bats of the species have 10–2000 viable *Histoplasma* cells in their faeces, when the total faecal contents were sampled.^27^

Further supportive evidence of past infection using *Histoplasma* antibody detection in bats was sought in several studies and negative in all samples.^25,27,50^ The immune response to natural infection by *Histoplasma* spp. in bats seems muted.^44^

### Isolation of *Histoplasma capsulatum* from soil and guano linked to bats

Indirect evidence of bat infection by *Histoplasma* spp. was sought by sampling and culturing guano from bat caves and roosts, and soil from these locations. In 10 studies, guano from the same bat species were found to be infected as from the bats themselves (Table 3). An additional 18 species of bats were presumptively infected, including four species of *Hipposideros*, based on guano alone (Table 3). Isolations of *H. capsulatum* were reported from multiple countries, including Puerto Rica, Thailand, Trinidad, Panama, Malaysia, Australia, Slovenia and the USA.

**Table 3.** *Histoplasma*-positive guano and soil from bat roosts implicating a particular species of bat as infected.

Species	Common name and additional data	Country	References
*Artibeus jamaicensis parvipes*	Jamaican fruit-bat prefers humid and tropical habitats but is also adapted to cloud forests and drier tropical areas	Puerto Rica	Torres-Blasini, 1966^51^
*Aselliscus stoliczkanus*	Stoliczka's trident bat found across SE Asia	Changmai, Thailand	Norkaew, 2013^52^
*Carollia perspicillata*	Seba's short-tailed bat found in moist evergreen and dry forests usually below 1 000 m elevation	Trinidad	Emmons, 1963^53^
*Chaetura brachyura*	Short-tailed swift bat found in a range of habitats including savanna and open woodland	Trinidad	Emmons, 1963^53^
*Chilonycterus rubiginosa fusca*	Horseshoe bat with a paleo-tropic distribution	Panama	Shacklette, 1962^7^; Klite, 1965^27^
*Desmodus rotundus*	Small leaf-nosed vampire bat native to the neotropics	Trinidad	Emmons, 1963^53^
*Eonycteris spleaea*	Cave nectar bat also called common nectar bat or lesser dawn bat. It is found in Bangladesh, Brunei, and in several small islands in Indonesia and SE Asia	Malaysia	Ponnampalam, 1963^54^
*Eptesicus fuscus dutertreus*	Big brown bat inhabiting cities, towns, and rural areas, but is least commonly found in heavily forested regions	USA	Emmons, 1958^55^
*Glossophaga soricina*	Pallas's long-tongued bat found in forest and rainforest	Trinidad	Emmons, 1963^53^
*Hipposideros armiger*	Great roundleaf bat found in South Asia, Southeast Asia, and China	Malaysia, Changmai, Thailand	Ponnampalam, 1963^54^; Norkaew, 2013^52^
*Hipposideros biclor*	Bicolored rounded leaf bat. It is found in Indonesia, Malaysia, the Philippines, Thailand, Timor-Leste. It inhabits caves rock crevices and tunnels among lowland forests	Malaysia	Ponnampalam, 1963^54^
*Hipposideros diaderma*	Diadem leaf-nosed or diadem roundleaf bat occurs in lowland rainforest and open woodland	Malaysia	Ponnampalam, 1963^54^
*Hipposideros lylei*	Shield-faced roundleaf bat found in China, Laos, Malaysia, Myanmar, Thailand and Vietnam	Changmai, Thailand	Norkaew, 2013^52^
*Micronycteris megalotis*	Little big-eared bat widely distributed in many habitats including wet and dry areas, evergreen and deciduous forests, and swamps	Panama	Klite, 1965^56^
*Miniopterus schreibersii*	Long fingered bat roosts in caves, rock clefts, culverts and caverns	New South Wales, Australia and Slovenia	Hunt, 1984^57^; Mulec, 2020^58^
*Mollosus ater (*Molossus *rufus*)	Black mastiff bat.	Trinidad	Emmons, 1963^53^
*Mollosus major*	Velvety free-tailed bat that occurs in Central and South America. Also found roosting in houses in urban areas	Trinidad	Emmons, 1963^53^
*Myotis chinensis*	Vesper bat found in China, Hong, Kong, Myanmar, Thailand and Northern Vietnam	Changmai, Thailand	Norkaew, 2013^52^
*Myotis muricola*	Wall-roosting mouse-eared bat, or Nepalese whiskered myotis roosts in many sites including curled-up banana leaves, limestone forests, hollow trees, rock shelters, artificial caves, mines and tunnels, and old buildings	Malaysia	Ponnampalam, 1963^54^
*Phyllostictus roostus*	Unknown	Panama	Taylor, 1962^59^
*Phyllostomus hastatus*	This species inhabits tropical regions of the Americas, and Trinidad and Tobago	Trinidad, Panama	Taylor, 1962^59^; Emmons, 1963^53^; Hasenclever, 1972^31^
*Pteronotus suapurensis*	Taxonomy changed. Mustached bat	Panama	Taylor, 1962^59^
*Rhinolphus luctus*	Great woolly horseshoe bat lives in large caves in forests, rocky outcrops, and semi evergreen forests	Malaysia	Ponnampalam, 1963^54^
*Saccopteryx bilineata*	Greater sac-winged bat native to the Central and South America	Trinidad, Panama	Emmons, 1963^53^; Klite, 1965^56^
*Taphozous melanopogon*	Black-bearded tomb bat found in caves in Guangxi and Guangdong, sometimes in large numbers. Also roosts in temples, ruins and other old buildings in India	Changmai, Thailand	Norkaew, 2013^52^
*Tadarida brasiliensis*	Mexican free-tailed bat	USA	McMurray 1982^50^

### Outbreaks of histoplasmosis associated with bat guano, implicating species of bats

An outbreak of histoplasmosis is defined as the occurrence of at least two cases of the disease. Numerous reports of epidemics or outbreaks of histoplasmosis in the Americas have been described over the years, dating back to the late 1930s.^19,60,61^ To our knowledge, only one of these reports identified the species of bats linked to exposure—in Costa Rica. In this report, 15 students entered a cave in Santa Rosa National Park, Guanacaste Province inhabited by about 500 bats.^62^ Twelve of the students subsequently developed persistent illness, with abnormal chest radiographs and serologic evidence of acute pulmonary histoplasmosis. The cave housed three species of fruit bats (*Glossophaga soricina, Carollia perspicillata*, and *Carollia subrufra*) and one species of vampire bats (*Desmodus rotundus*).

Staffolani et al. (2018) in an exhaustive review of literature identified 814 persons who acquired acute or disseminated histoplasmosis amongst the travellers to bat caves in different countries in the Caribbean, South America, some parts of North America, Europe, and Africa.^63^ These authors also mentioned details of exposure to bats, cases occurring in clusters, and types of antifungal therapy if given. They also provided a timeline distribution of the studies reporting cases of acute histoplasmosis in immunocompetent travellers.

## Discussion

In 1981, Hoff and Bigler counted 24 species of bats with documented *Histoplasma* infection recorded (two not confirmed).^18^ We identified an additional 25 bat species infected, with indirect evidence of infection of another 18 species (total of 67). There are over 1400 species of bats present worldwide. The vast majority of studies of *Histoplasma* infection in bats are from the Americas, including the Caribbean, with a few others from Thailand, Malaysia, Australia, Slovenia, France, Iran, DRC, South Africa, Tanzania, and for *Histoplasma duboisii* in Nigeria. Additional hyperendemic areas of histoplasmosis (based on histoplasmin skin testing population frequency) include Myanmar, southern Thailand, parts of Java Island in Indonesia, Luzon in northern Philippines, and probably other areas of SE Asia.^64^ It is highly likely that these and other areas have resident and migratory bat populations with histoplasmosis. Not only are bat and environmental data on *Histoplasma* missing from these and other areas, but reports of outbreaks of acute pulmonary histoplasmosis from most of these areas are also absent, the exceptions being New Caledonia, Indonesia, and South Africa.^63^

Taxonomy of the *Histoplasma* genus has recently undergone a revision with *H. capsulatum* var *capsulatum* being split into four species: *H. capsulatum sensu stricto* (also known as the Panama or H81 lineage), *Histoplasma ohiense, H. mississippiense*, and *H. suramericanum*.^65^ This taxonomic revision has yet to be fully accepted. It will take some time to ascertain if certain bat species are more or less likely to harbour any of these species/varieties/taxonomic groups preferentially. One bat from Sao Paolo state in Brazil was infected with *H. suramericanum*, assuming this is a valid species name.^66^ Recently sequence data from a small number of *H. capsulatum* isolates from bats in Mexico and migratory bats has started to unravel these relationships.^36^

While bats may have *Histoplasma* spp. isolated from deep tissues, notably lungs, liver and spleen, and may be experimentally infected with low inocula, it is not clear to what extent they suffer from disease, or whether they have latent infection, with gastrointestinal colonization or excretion. Pathologic lesions were seen a few examined specimens of *Pteronotus* (*Chilonycteris) rubiginosa*, but not many. It is probably unwise to generalize from one species to all others, especially where attempts to isolate *Histoplasma* spp. from multiple other bats was unsuccessful (Supplementary Table 1). Likewise given the variable manifestations and yield from these prior experiments (which often used mouse inoculation to improve culture sensitivity), it would be unwise to assume that some species of bat were intrinsically immune to infection with *Histoplasma* spp. A complicating factor in understanding *Histoplasma* infection in bats is their variable temperature over time. There is probably some variation in temperature in different species, but body temperature during flight is typically 40°C, and it falls to lower than 10°C when in torpor.^67^ These temperatures bracket the dimorphism temperature sensitivity of *Histoplasma* spp, typically around 32°C, when filamentous forms at the lower temperature shift to a yeast form. To add further complexity, some strains of *H. capsulatum* grow more slowly at higher temperature, notably above 39°C.^68^ To our knowledge the biological implications of the temperature dynamics of bats on the physiology of *Histoplasma* spp. have yet to be fully addressed. Changing weather patterns, extremes of temperature and possibly altered migratory patterns of bats in response to these climatic pressures, could alter the ‘status quo’ between bats and *Histoplasma* spp..

The recent outbreak of the lethal fungal infection of bats in N. America of White Nose Syndrome,^69–71^ has led to a resurgence of interest in bat disease. Further attempts are underway to immunize bats to prevent mass die-offs and potentially species extinctions.^72^ It is conceivable that *Histoplasma* infection of bats could evolve and become problematic for bats, humans and the other mammals that can be infected. Infection due to *H. capsulatum* in cats and dogs is reported to be frequent in Midwest and southern United States.^73^ A recent survey of *Histoplasma* in mammals in Europe indicates a much wider endemic area than previously appreciated.^74^

## Conclusion

This study provides an update on the occurrence of *Histoplasma spp*. in bats and soil mixed with bat guano. Fifty four of the approximately 1400 species of bat have been documented to be infected by *Histoplasma* spp. There has been slow progress in this area in recent decades. There is a need to explore the occurrence of histoplasmosis in human populations around caves that harbour bats in areas of endemicity of histoplasmosis. Only one bat species has been reported to be infected with *H. duboisii* (in Nigeria), yet this fungus is present throughout the African continent. A better understanding of the pathogenicity, latency, and intestinal excretion of *Histoplasma* in bats is called for.

## Supplementary Material

myad080_Supplemental_File
