# Supplementary material for: Infection of bats with Histoplasma species
Source: Med Mycol. 2023 Aug 8;61(8):myad080. doi: 10.1093/mmy/myad080 (PMC10802898; doi:10.1093/mmy/myad080)
Supplement: myad080_Supplemental_File [file myad080_supplemental_file.docx]

**Gugnani and Denning**

Supplementary table 1. Occurrence of *Histoplasma capsulatum* var. *capsulatum* in soil, soil admixed with bat guano, bat guano/chicken excreta, or in bats in some countries.

| **Country** | **Location** | **Type of samples** | **No. pos. /no examined (if known)** | **Method of Detection** | **Reference** |
| --- | --- | --- | --- | --- | --- |
| Venezuela | Lara State | Soil from cave housing bats | 4/5 (80%) positive | Culture of cave soil | Campins *et a*l. 1956 |
| South Africa | Johnson’s pothole, Hennop’s river, Transvaal | Soil and respiratory filters | Tingo Maia - 6/40 positive and in Cusco – 1/20 positive | Direct culture and mouse inoculation | Murray et al 1957 |
| Tanzania |  | 13 soil samples from a bat inhabited cave | 7/13 samples positive. | Mouse inoculation of soil filtrate | Ajello, 1960 |
| Trinidad |  | Hollow trees and 1 bat inhabited cave | 4/14 samples positive | Mouse inoculation of soil filtrate | Ajello, 1960 |
| Republic of Panama | Building at Madden Airfield | 100 bats and guano and soil, 14 *Chilonycterus rubiginosa fusca* and 45 *Carollia perspicillata* bats | *Carollia perspicillata (0/45) and Chilonycterus rubiginosa fusca* (9/14) and soil positive | Culture of pools of homogenized liver and spleen, and mouse inoculation. Soil and guano refrigerated and cultures done and then inoculated into mice. | Shacklette et al, 1962 |
| Panama Canal Zone | Chilibre Cave | Soil and bat guano Five bats identified as *Phyllostictus roostus* | Top and subsurface soil samples, soil samples from animal burrows and their nests decaying wood, waste from leaf-cutter ant gardens 14/426 (3.3%) positive | 2 gm of soil in antibiotic-treated saline was inoculated intraperitoneally into mice. Homogenized pieces of liver and spleen sacrificed (at intervals of 2-5 wks.) were cultured. Cultures were examined over 6 wks. | Taylor et al 1962 |
| Trinidad |  | Houses, buildings and trees inhabited by bats | 8/176 soil samples positive | Mouse inoculation of soil filtrate | Emmons, 1963 |
| Malaysia | Bat cave north of Kuala Lumpur | Soil samples. Cave houses *Eonycteris spleaea, Hipposideros diaderma, H. biclor, H. armiger, Myotis muricola* and *Rhinolphus luctus* | 1/5 (20%) soil samples positive | Soil inoculated into mice, with death of the mouse and subsequent culture of liver and spleen. | Ponnampalam, 1963 |
| Mexico | Soil from 13 states, some linked to caves | Soil investigations related to bat guano | *Tararida brasilensis* and *Desmodus rotundus* | Soil culture and outbreak investigations | Gonzalez-Ochoa, 1963 |
| Panama | Curunda (near Panama City) | Soil from a bat roost in a tree and live bats | 3/10 (33.3%) guano samples positive - *Saccopteryx bilineata* (0/3) and *Micronycteris megalotis* (3/7) | Direct tissue culture of liver, spleen and lungs, and soil samples. | Klite and Young, 1965 |
| Panama | 31 sites | Live bats captured during the day | *Artibeus cinereus* (0/5)*, A. jamaicencis (*0/15), *A. literatus* (0/1), *Carollia perspicillata (*7/141), *Chilonycteris rubiginosa* (30/120), *Glossophaga soricine* (2/57), *Lonchohina, aurita* (0/18), *Micronicteris megalotis* (19/84), *M. nicefori* (0/3), *Molossus major* (1/99), *Myotis nigricans* (0/29), *Phyllostomus hastatus* (3/19), *Prompos centralis* (0/6), *Saccopteryx bilineata* (0/23), *Uroderma bilobatum* (0/3) positive*.* Antibodies were not detected, including 8 bats with positive cultures. Intestinal histopathology positive. | Bats frozen at -20C for 2-6 weeks. Liver, spleen, lung and kidney macerated and cultured. Intestinal contents processed separately (after removal of organs). Intestinal contents and liver/spleen pools were inoculated into mice. Sera from 40 bats were analysed for *Histoplasma* precipitating antibodies. Histopathology of the intestine in *Chilonycteris rubiginosa.* | Klite and Diercks, 1965 |
| Panama | Multiple locations | 935 live bats | *Carollia perspicillata azteca 1/246* (0.4%), *Chilonycteris rubinosa fusca* 6/200 (3%), *Desmodus rotundus* 2/9 (22%), *Glossphaga sorcina* 1/33 (3%) and *Lonchorhina aurita aurita* 1/20 (5%), *Carollia subrufa* 0/2*, Carollia spp.* 0/25*, Artibeus cinerus* 0/20*, A. jamaicensis* 0/122*, A. lituratus palmarum* 0/12*, Chiroderma salvani* 0/6*, Lonchophylla robusta* 0/2*, Macrophyllum macrophyllum* 0/2*, Micronyteris megalotis* 0/1*, Molossus bondae* 0/2*, M. coibensis* 0/43*, olossus sp.* 0/29*, Myotis nigicans* 0/15*, Peropteryx kappleri* 0/3*, Phyllostomus hastatus panamensis* 0/2*, Pteronotus suapurensis* 0/52, *Saccopteryx bilineata bilineata* 0/3, *Sturnira lilium lilium* 0/3, *Trachops cirrhosis* 0/1, *Uroderma bilobatum* 0/31*, Vampyrodes major* 0/10*, V. helleri* 0/2, *V. vittatus* 0/8 *and Vampyrum spectrum nelson* 0/1*.* | Liver and spleen emulsions cultured | Diercks, 1965 |
| El Salvador | La Libertad | 72 bats | *Phyllostomus discolor* (1/6), *Chilonycteris rubiginosa* (0/2), *Glossophaga socicina* (0/12) and *Artibeus jamaicensis* (10/52) | Bats frozen at -20C for 2-6 weeks. Liver, spleen, lung and kidney macerated and cultured. Intestinal contents processed separately (after removal of organs). Intestinal contents and liver/spleen pools were inoculated into mice. | Klite, 1965 |
| Bolivia | San Joaquin | 187 bats of *Malosus major, M. atea, Eumops bonariemsis, E. perotis, Myotis nigicans, Noctilio labialis* | All negative | Bats frozen at -20C for 2-6 weeks. Liver, spleen, lung and kidney macerated and cultured. Intestinal contents processed separately (after removal of organs). Intestinal contents and liver/spleen pools were inoculated into mice. | Klite, 1965 |
| USA | Maryland and Frio Cave, Concan Texas | 111 *Eptesicus fuscus* and 500 *Tadarida brasiliensis. Soil and Guano* | *Eptesicus fuscus* (1/111) and *Tadarida brasiliensis* (50/302) were positive. Different organs were positive, notably lungs in *T. brasiliensis.* Soil but not guano from Maryland was positive. | Liver, spleen, lung, kidney and sections of intestines were cultured and processed for histopathology. | Emmons, 1966 |
| Puerto Rica | Soil samples (mostly top soil) from various localities | 120 soil samples from a cave housing *Artibeus jamaicensis* | Soil samples from two different places in the cave positive | Inoculation of soil suspensions into mice, and histopathology of sacrificing them for histopathology of internal organs and their culture | Torres-Blasini and Carrasco-Canales 1966 |
| Isthmus of Panama | Limestone cave on the shore of Maden Lake | Studies on bats, soil, and 27 air and samples: *Pteronotus rubiginnosa*. | The first isolation of *H. capsulatum* from this species. | Methods not described: | Shacklette *et al.* 1967; Hasenclever, 1969 |
| USA | Alabama University attic | 77 Tadarida cynocephala and guano | Tadarida cynocephala 13/77 and 2/42 guano samples positive |  | Ajello, 1967 |
| USA | Many states | 1120 bats of 8 species | 154/1120 of *Myotis austroriparius, M. grisescens, M. sodalis* positive | Bats frozen at -20C for <5 weeks. Liver, spleen, lung and intestinal contents cultured. | Tesh *et al*, 1967 |
| Colombia (Western and Central Region) | 1001 bats of neotropical species from 12 sites in collected b/w 1966-1967 | Live bats of 31 different species | *Carollia perspicillata* (1/173), *Eptescicus brasiliensis* (1/1) *Desmodus rotundus* (1/85) positive | Cultures of lungs, liver and spleen of bats on mycological media | Tesh *et al*. 1968 |
| USA | Cave occupied by bats in Oklahoma | 50 *Tadarida brasiliensis bats* | *Tadarida brasiliensis* (6/50) | Liver, spleen and lung and macerated and cultured. | Bryles, 1969 |
| Panama | 13 sites in abandoned houses trees, caves | 2424 live bats of 21 species. Air and soil samples Mice and hamsters used as sentinels. | *Artibeus spp,* (0/67), *Carollia perspicillata* (8/652), *Desmodus rotundus* (1/31), *Glossophaga soricina* (2/73), *Lonchophylla robusta* (4/24), *L. aurita* (1/4), *Macrophyllum macrophyllum* (0/31), *Micronycteris megalotis* (0/2), *Mimon crenulatum* (0/1), *Molussus* sp. (1/111), *Natalus natalus* (0/7), *Noctolio labialis* (5/8), *Phyllostomus discolor* (2/6), *P. hastatu*s (22/118), *Pteronotus rubiginosa* (283/1124), *P. suapurensis* (4/33), *Sarcopteryx bilineata* (0/4), *Tadarida yucatanica* (3/89), *Tonatia bidens* (1/7), *Uroderma bilobatum* (0/29) and *Vampynops helleri* (0/3) positive. Air (n=1), water (n=1) and soil (n=4) samples were positive by culture. | Liver, spleen, lung and kidney macerated and cultured. Intestinal contents processed separately (after removal of organs). Air, water and soil samples were filtered and inoculated into mice. | Shacklette, Hasenclever, 1969 |
| Panama | Limestone cave on the shore of Maden Lake | 109 *Pteronotus rubiginnosa,* 14 *Carollia perspicillata* and 5 *Phyllostomus hastatus* bats | *Phyllostomus hastatus* (1/5)*, Carollia perspicillata* (0/14) *and Pteronotus rubiginnosa* 38/109 positive by culture and 40/109 *Pteronotus rubiginnosa* positive microscopically | Liver, spleen, lung, kidney and sections of intestines were cultured and processed for histopathology. | Hasenclever, 1969 |
| USA | 14 sites in south Arizona | 555 live bats of 9 species | *Tadarida brasiliensis Mexicana (8/251), Leptonycteris sanborni (7/134)* | Liver, spleen and lungs were cultured. Histopathology was done on liver, spleen and intestine. Soil and guano cultured, Bat fur cultured for dermatophytes. | Di Salvo, 1969 |
| Canal Zone and surrounding Panama Republic | Three different sites | Bats of different species | 389/2234 (17.4%) positive  *Carollia perspicillatus* 8/652 (1.2%)  *Glossophaga soricine* 2/73 (2.7%)  *Mollosus* sp 1/111 (0.9%)  *Phyllostomus hastatus* 22/118 (18.6%)  *Pteronotus rubiginosa* 283/1124 (25.2%)  *Tadarida yucatanica* 3/89 (3.3%)  *Aribeus* spp. 0/67 | Bats were sacrificed and 10 cm portions of each bat was cultured on mycological media Tissues of internal organs were also examined histologically.  Sampling of soil, air, and water at six sites was positive for soil from 3 sites, air and water from one site each. Culture was more sensitive than microscopic examination of tissue sections in in organs other than the intestine. | Hasenclever et al 1972 |
| Zaire |  | Captured bats | 0/87 of ‘megachiropters’ and ‘microchiropters’ positive | Lungs, livers, spleens and intestinal contents cultured, some after freezing at -20C. | De Vroey, 1972 |
| Cuba | Caves ‘Luis Torres’ and ‘Cueva Grande’ in Havana Province | 169 bats of 5 species | *H. capsulatum* was isolated from 4 of the five species: *Artibeus jamaicensis* *parvipes* (8/75), *Brachyphylla nana* (5/29)*, Eptesicus fuscus* *dutertreus* (2/16), *Tadarida brasiliensis muscula* (0/48) and *Mormoops blainvillel* (1/1) All bats were serologically negative for *H. capsulatum.* | Bats were sacrificed and blood collected. Liver, spleen and lungs were collected, homogenized, and cultured for up to 3 months. Sera were analysed by immunodiffusion. | D'Escoubet and Olano 1976. |
| Belize | San Antonio cave, Maya Mountain cave | Soil, 26 bats of the species - *Carolli brevicauda* (3)*. C.* perspicillatus (1), *Desmodus* rotundus (3), *Glossophaga soricine* (10), *Micronycteris bardyotis* (2), *Myotis* sp. (1) *Pteronotus parnellii* (6) | 0/20 soil specimens, 1/6 (17%) *Pteronotus parnellii* | Culture of soil. Bats were frozen and transported to CDC, thawed and liver, spleen and lung cultured. | Quinones *et al.* 1978 |
| Puerto Rica | Aguas Buenas caves in Barrio Sumidero | Soil and air samples | Only one of 19 (5.2%) soil sampling sites yielded *H. capsulatum* | Isolation of *H. capsulatum* from soil samples was attempted by culture. Air samples were collected by portable Venturi scrubber air samplers into distilled water. Using a Goetz Milli-pore filter, water was injected into mice with subsequent culture of their lungs, liver and spleen. | Carvajal and Zamora 1977a and 1977 b. |
| USA | Bracken Cave, San Antonio, Texas | Soil, guano and live bats. Mouse exposure in cave. | 8/14 (57%) of soil samples positive. 0/28 *Tadarida brasiliensis* bats. 0/58 bat sera were positive. 1/5 (20%) exposed mice positive | Culture of soil. Culture and histopathology of lung, spleen and liver. Antibody measurement of bat sera. | Murray and Russell, 1982 |
| Cuba | Island of Juventad | Soil from caves with 10 species of bats present | 5/30 (17%) in Cocodrillos cave, 11/30 (37%) in Pedernales cave positive | Direct microscopy, culture and mouse inoculation | Fernandez Andreu and Martinez-Mashin1984 |
| Australia | Wee Jasper, New South Wales | Soil, bat guano, bat carcasses | 2 samples positive from a single visit of 6 visits over 7 years. | Culture, mouse inoculation and liver, spleen and lungs cultures, and mice exposed to the cave environment | Hunt et al 1984 |
| Jamaica |  | Soil inside caves | 1 positive sample. | Culture done by Libero Ajello | Fincham 1998 |
| Mexico | Guerrero and Morelos | 208 bats of 18 different species | 1/16 (6%) *Pteronotus parnellii,* 1/3 (33%) *Myotis californicus,* 1/7 (14%) *Mormoops megalophylla,* 2/5 (40%) *Natalus stramineus,* 10/15 (67%) *Artibeus hirsutus* and 2/9 (22%) *Leptonycteris nivalis* positive | Histopathology and culture of guts, liver, spleen and lungs, with addition of *Histoplasma* growth factor to cultures. | Taylor et al, 1999 |
| Iran | 2 caves in North (Lahidjan) and West (Kermanshah) | 800 bats representing three genera, viz. *Myotis* spp.*, Rhinoloptus* spp., *Sherbersi* spp. were examined in 2 caves in the North and West in Iran. | *H. capsulatum* was not isolated from any of the 800 bats. | The bats were autopsied and their lungs, liver, kidney, spleen and intestinal contents were examined by direct microscopy in KOH and Geinsa stain, histopathology, and culture on mycological media at 20^0^C and 37^0^C. Internal organs of tissue samples of 100 were inoculated into mice intraperitoneally. | Hashemi, 2003 |
| Argentina | Morón, Buenos Aires | Soil mixed with pigeon and bat droppings | 5/5 (100%) positive | Intraperitoneal inoculation of hamsters | Negroni et al 2010 |
| Mexico and Argentina | Chiapas (La Trinitaria cave), Michoaca ́ n (Isla de Janitzio cave), Hidalgo (El Salitre cave), Nuevo Leo ́ n (La Boca cave), Tucuma ́ n (Dique Escaba grotto) and Co ́ rdoba (Cemetery tunnel) | 87 live migratory bats | 71/87 (82%) *Tadarida brasiliensis* | Bat lungs and PCR | Gonzalez- Gonzalez et al 2012 |
| Mexico, Brazil and Argentina | *H. capsulatum* isolate collection | Randomly captured bats | *Artibeus hirsutus* (n=9), *Leptonycteris nivalis* (n=6), *L. curasoae* (n=8), *Desmodus rotundus* (n=1), *Pteronotus davyi* (n=1), *Tadarida brasiliensis* (n=9), *Molossus rufus* (n=1), *M. molossus* (n=9) and *Eumops bonariensis* (n=1) | Cultures from bats (organs and methodology not specified, then identified and typed using micro-satellites). | Taylor, 2012 |
| Thailand | Chaingmai | Soil admixed with bat guano | 7/88 (8%) positive | Nested PCR | Norkaew *et al.* 2013 |
| France | La Palmyre zoo, Bourges | 83 dead bats | *Nyctalus noctule* (1/18), *Rousettus aegyptiacus* (0/16), *Pteropus rodricensis* (0/45), *Plecotus austriacus* (0/1) and *Pipistrellus pipistrellus* (0/3) | Nested PCR from lung samples | Gonzalez- Gonzalez et al 2013 |
| Argentina, French Guyana, Mexico | 122 randomly captured bats of different species. | Argentina 21 bats French Guyana 13 bats,  Mexico 88 bats | 98/128 (77%) positive. 43 bats were coinfected with *Pneumocystis* | The lungs from bats captured from MX were frozen at -20^0^C. Bats from AR and FG had their lungs were preserved in 70% alcohol. The presence of *H. capsulatum* in the lungs of bats was tested by nested PCR. | Gonzalez- Gonzalez et al 2014 |
| Brazil | 249 bats from Mato Grosso and Rio Grande do Sul | Live bats captured in caves, forests, and urban areas | 4 bats were not identified and were negative. *Artibeus fimbriatus* (5/7, 71%), *A. lituratus* (2/3), *Desmodus rotundus* (13/50, 26%), *Histiotus velatus* (12/36, 33%), *Molossus molossus* (7/30, 23%), *M. rufus* (2/5), *Tadarida brasiliensis* (13/45, 29%), *Eumops glaucinus* (1/6), *Glossophaga sorcina* (1/8), *Lasiurus blossevillii* (1/4), *Myotis levis* (1/6), *M. nigricans* (1/2), *Nyctinompos laticaudatus* (1/22), *N. macrotis* (1/2) positive. *Carollia perspcillata* (0/2), *Diaemus yoingu* (0/1), *Diphylla eucadata* (0/1), *Eptesius furinalis* (0/1), *Lasiurus cinereus* (0/1), *Molossus correntium* (0/1), *Rhinophylla puimilio* (0/1) and *Sturnira lilium* (0/1). | Lungs (250g) by PCR, after manual kit DNA extraction | Veloso, 2014 |
| Brazil | Twelve municipalities in the Midwest region of the state of São Paulo | 172 bats (species not known) | 12/172 (7%) positive | Presence of *H. capsulatum* in bats tested by nested PCR and culture of liver, spleen, and lung samples. | da Paz et al 2018 |
| Brazil | South-eastern Brazil | 89 bats | 31/89 positive, 12 species not identified. *Molossus molossus* (8/21, 38%), *Molossus* spp. (5/11, 45%), *Eumops* spp. (1/4), *Eumops glaucinus* 1/3), *Eumops perotis* (2/6), *Artibeus literatus* (2/6), *Platyrrhinus lineatus* (1/1) positive. *Molossus rufus* (0/2), *Eumops auripendulus* (0/1) *Tadarida brasiliensis* (0/1), *Nyctinomops* spp. (0/1), *Artibeus obscurus* (0/1), *Artibeus* spp. (0/4), *Platyrrhinus* spp. (0/1), *Pygoderma bilabiatum* (0/1), *Carollia perspicillata* (0/1), *Desmodus rotundus* (0/1) *Myotis* spp. (0/5), *Histiotus montanus* (0/1) negative | Lung samples with automated DNA extraction and PCR | Dos Santos, 2018 |
| Slovenia | Bat caves from five locations | Bat guano | 6/6 (100%) positive | Antigen enzyme immunoassay positive, PCR negative | Mulec *et al*. 2020 |
| Brazil | 18 cities in Para state, one forested area and one town |  | *Carollia perspicillata* (1/40) and *Vampyriscus bidens* (1/1) positive; *Artibeus obscurus* (0/1)*, Artibeus cinereus* (0/1), *Artibeus (dermanura)sp.* (0/1), *Artibeus planirostris* (0/14), *Glossophaga soricina* (0/6), *Hsunycteris thomasii* (0/4), Micronycteris minuta (0/1), *Phyllostomus discolor* (0/4), *Phyllostomus fusiventris* (0/1), *Rhinophylla pumillo* (0/3), *Sturnira lilium* (/2), *Uruderma bilobatum* (0/1), *Uruderma magnirotrus* (0/2), *Gardnerycteris crenulatum* (0/2), *Eumops auripendulus* (0/3) *Eumops glaucinus* (0/1), *Molosus molosus* (0/7), *Molosus rufus* (0/2), *Natalus macrourus* (0/1). | Cryopreserved liver and lung samples and paraffin blocks from heart, lung, liver, spleen, skin and muscle with PCR | Da Silva, 2020 |
| Ciudad de México |  | Randomly captured bats (species not in danger of extinction) | Only bats with positive cultures are described; 9 *Tadarida brasiliensis* and 4 *Leptonycteris nivalis* | Bats were processed for detection of *H*. *capsulatum* using PCR. A concentrated sequence-types network nucleotide diversity analysis provided found a species-level clade containing seven *H*. *capsulatum* from the bats. | Vite-Garin et al 2021 |
